# Role of the Anterior Center-Edge Angle on Acetabular Stress Distribution in Borderline Development Dysplastic of Hip Determined by Finite Element Analysis

**DOI:** 10.3389/fbioe.2022.823557

**Published:** 2022-03-01

**Authors:** Songhao Chen, Liqiang Zhang, Yuqian Mei, Hong Zhang, Yongcheng Hu, Duanduan Chen

**Affiliations:** ^1^ School of Life Science, Beijing Institute of Technology, Beijing, China; ^2^ Tianjin Medical University, Tianjin, China; ^3^ Department of Orthopaedics, Shanxi Children’s Hospital, Taiyuan, China; ^4^ School of Medical Imaging, North Sichuan Medical College, Sichuan, China; ^5^ Department of Orthopaedics, The Fourth Medical Centre of PLA General Hospital, Beijing, China; ^6^ Department of Bone and Soft Tissue Oncology, Tianjin Hospital, Tianjin, China

**Keywords:** borderline developmental dysplasia of the hip, cartilage of hip joint, finite element analysis, stress distribution, hip joint

## Abstract

**Background:** The joint with hip dysplasia is more likely to develop osteoarthritis because of the higher contact pressure, especially in the socket. The lateral center-edge angle (LCEA) is the major indicator for hip dysplasia *via* radiography. However, the pathological conditions of LCEA angles in the range of 18°–25° are still controversial, which challenges precise diagnosis and treatment decision-making.

**Objective:** The purpose of this study is to investigate the influence of anterior center-edge angle (ACEA) on the mechanical stress distribution of the hip joint, *via* finite element analysis, to provide insights into the severity of the borderline development dysplasia.

**Methods:** From 2017 to 2019, there were 116 patients with borderline developmental dysplasia of the hip (BDDH) enrolled in this research. Based on the inclusion criteria, nine patients were involved and categorized into three LCEA groups with the maximal ACEA differences. Patient-specific hip joint models were reconstructed from computed tomography scans, and the cartilages, including the labrum, were established *via* a modified numerical method. The finite element analysis was conducted to compare the stress distributions due to the different ACEA.

**Results:** As ACEA decreased, the maximum stress of the acetabulum increased, and the high stress area developed toward the edge. Quantitative analysis showed that in the cases with lower ACEA, the area ratio of high stress increased, and the contact facies lunata area significantly affected the stress distribution.

**Conclusion:** For patients with BDDH, both the ACEA and the area of facies lunata played essential roles in determining the severity of hip dysplasia and the mechanical mechanism preceding osteoarthritis.

## 1 Introduction

The hip joint is the most critical structure for stability and weight-bearing in the joint formation between the acetabulum and the femoral head (Zhao et al., 2010). Developmental dysplasia of the hip (DDH) is a common orthopedic disease and a complex developmental disorder. The anatomical features usually manifest as incomplete wrap of the acetabulum into the anterior and lateral parts of the femoral head, reducing the coverage area of the contact in the hip joint (Murphy et al., 1990). This disease can lead to structural instability of the hip joint and thus result in osteoarthritis (Klaue et al., 1991; Weinstein et al., 2003). From a biomechanical perspective, the reduction in load transfer area contributes to the increased contact pressure distribution in the dysplastic hip joint in daily life (Russell et al., 2006; Henak et al., 2013). Pathologically, because of the cumulative effect of time in the areas of high stress concentration and the discontinuous distribution over articular cartilage (Henak et al., 2014), it will eventually cause acetabular labrum and cartilage damage, accelerating the hip degradation (Murphy et al., 1995).

The diagnosis of DDH mainly relies on the structural characteristics of the hip joint based on X-ray image measurements (Chosa et al., 1997). Wiberg initially proposed an index named the lateral center-edge angle (LCEA), which is the angle formed by a line connecting the center of the femoral head perpendicular to the transverse axis of the pelvis and the line connecting the center of the femoral head and the uppermost lateral point of the acetabular sclerosis weight-bearing area in the anterior and posterior view of the pelvis in standing position (Wiberg, 1939). According to this definition, LCEA <18° is defined as hip dysplasia, whereas LCEA >25° is defined as the normal state. However, when LCEA is between 18° and 25°, the pathological status of the hip is uncertain and is defined as critical dysplasia. A few researches defined the hip with the LCEA in the range of 18°–25° as the borderline developmental dysplasia of the hip (BDDH) (Domb et al., 2013; Chandrasekaran et al., 2017; Hatakeyama et al., 2018) and indicated that the hip joint under this condition is in a state of mild dysplasia (Fukui et al., 2015; Matsuda et al., 2017; Ricciardi et al., 2017). Recent researches also suggest that the LCEA alone might not be adequate to evaluate the severity of hip dysplasia (Jessel et al., 2009; Fujii et al., 2010). Multiple morphological features, such as the anterior center-edge angle (ACEA), the acetabular wall indices (Siebenrock et al., 2012), the femoral epiphyseal acetabular roof, and the Tönnis angle (Lequesne and de SEZE, 1961), should be involved. In particular, the ACEA can assess the anterior coverage of the femoral head, which can significantly affect the magnitude and distribution of stress in the hip joint (Lequesne, 1961a; Ganz et al., 2003). The parameter is the angle formed by a vertical line passing through the center of the femoral head and the line passing through the center point of the femoral head and the most anterior point of the acetabular sourcil on a pseudosection radiograph.

With the rapid development of interdisciplinary research in the medical field, numerical computing technology has made significant contributions to medical problem analysis and risk prediction in the study of pathological mechanisms. Finite element analysis (FEA) is an effective tool to reveal biomechanics (Kaku et al., 2004) and has been widely applied to investigate the biomechanical behavior of spine and tibia bone with good agreement with experimental measurements (Dalstra et al., 1995; Anderson et al., 2008; Anderson et al., 2010). Tan et al. conducted numerical study of bone healing process of a transverse fractured tibia (Tan et al., 2021). Kosalishkwaran et al. applied this technique to the spine, proposing an innovative method for calculating RoM (Kosalishkwaran et al., 2019). Furthermore, Schuller et al. first used the FEA technique to study the dysplasia of the hip (Schuller et al., 1993), and the subsequent researches mainly focused on the mechanical evaluation of different LCEAs and Bernese periacetabular osteotomy treatment.

In this study, specifically targeting the BDDH, we aimed to investigate the mechanical influence of ACEA by establishing detailed models of the hip joint, including the acetabulum, the femoral head, the acetabular cartilage, and the femoral head cartilage. The spatial stress distributions of the acetabulum and acetabular cartilage were analyzed in detail to assist the clinical diagnosis of the severity of hip dysplasia.

## 2 Methods

### 2.1 Patients

This retrospective research collected 116 patients (144 hips) diagnosed with BDDH in the Fourth Medical Center of PLA General Hospital from July 2017 to May 2019 at the outpatient service. Informed consent was obtained for the studies with human subjects.

For the purpose of this study, inclusion criteria included the following: (1) the LCEA was in the range of 18°–25°; (2) the ACEA was less than 25°; (3) the pseudosection radiographs and standing anteroposterior pelvic and computed tomography (CT) scanning data were well preserved; (4) the Tönnis osteoarthritis grade less than or equaled to 1. The patients were excluded according to the following: (1) the patients had an operation history of the hip disease; or (2) the patients had a medical history of neuromuscular diseases, such as cerebral palsy or poliomyelitis.

**FIGURE 1:**
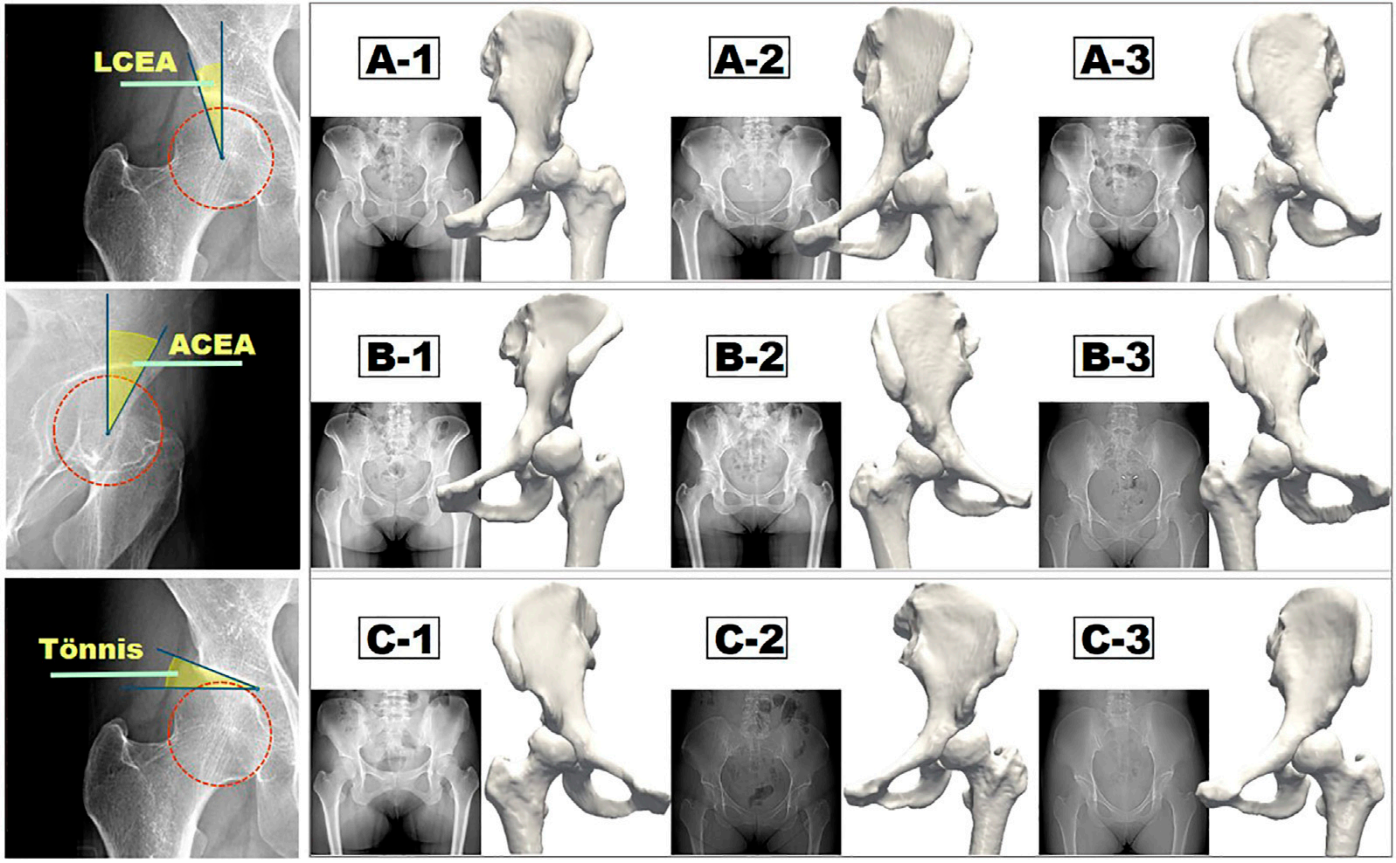
Measurement methods of three major morphological indices (LCEA, ACEA, and Tönnis angle) and the corresponding three groups of reconstructed hip joint models.

Eventually, nine hips with borderline dysplasia were included in our study. The study was approved by the institutional review board. All enrolled patients who agreed to participate in this study and publish the results of the study signed an informed consent form. The average age of patients was 35.33 years (range = 24–48 years), and the relevant patient information is listed in Table 1. The LCEA, ACEA, and Tönnis angle were all measured three times by two experienced orthopedic surgeons independently, and the averaged values were set as the ultimate results. As the ACEA is changing with the LCEA in the same hip joint model, the use of idealized models to form different LCEAs and ACEAs by rotating the acetabulum and femoral head is not applicable to this study. The ACEA greater than 25° was considered as a physiological condition that was excluded in this research. Eventually, as shown in Figure 1, nine hips with the available LCEAs, ranging from 18° to 20°, satisfying the condition of ACEA <25°, were divided into three groups (A, B, C) with various ACEAs with a maximal difference. Because of the interdependent relationship between LCEA, ACEA, and Tönnis angle, the otherness of Tönnis angle was not considered temporary in this research.

**TABLE 1:** Clinical data of patients with borderline developmental dysplasia of the hip.

Patients	A_1	A_2	A_3	B_1	B_2	B_3	C_1	C_2	C_3
Hip affected	Left	Left	Right	Left	Right	Right	Right	Left	Left
Gender	Female	Female	Female	Female	Female	Female	Female	Female	Female
Age (years)	35	34	24	31	42	41	39	48	24
LCEA (°)	18.1	18.1	18.2	18.9	19.1	19.1	20.1	20	19.9
ACEA (°)	21.6	15.7	13.9	24.8	19.8	−7.5	21	18.5	12.8
TONNIS (°)	10.6	15.2	11.3	10.8	13.5	11.1	11	15	13.1

### 2.2 Construction of Three-Dimensional Model of Hip Joint

The patient-specific three-dimensional (3D) models of the hip joint (bony structure, i.e., acetabulum and femur) were constructed from the CT cross-sectional images (GE Medical Systems, United States). Scanning parameters were as follows: voltage: 120 kV; slice thickness: 1.3 mm; kilovolt peak (kVp): 120 kVp; spacing between slices: 5 mm. The DICOM data of enrolled hips were loaded into the Mimics (Materialise, Belgium), and regions of femur and acetabulum were extracted by thresholding, region growing, and manual editing. Surgeons confirmed the final mask, and reconstructed 3D models were carefully improved by executing the smooth operation to reconstruct the original surface profile.

Cartilage structure and cartilage interface are significant factors in computing the stress distribution of the hip joint (Anderson et al., 2005). Currently, the commonly used techniques for cartilage construction include spherical cartilage modeling, uniform thickness cartilage modeling, and midline cartilage modeling (Anderson et al., 2005). The contact stress of the hip joint predicted by the first spherical cartilage modeling method is continuous, and the peak value is lower, which is closer to the physiological state (Li et al., 2018). For this reason, the spherical cartilage modeling method was used in this research.

**FIGURE 2:**
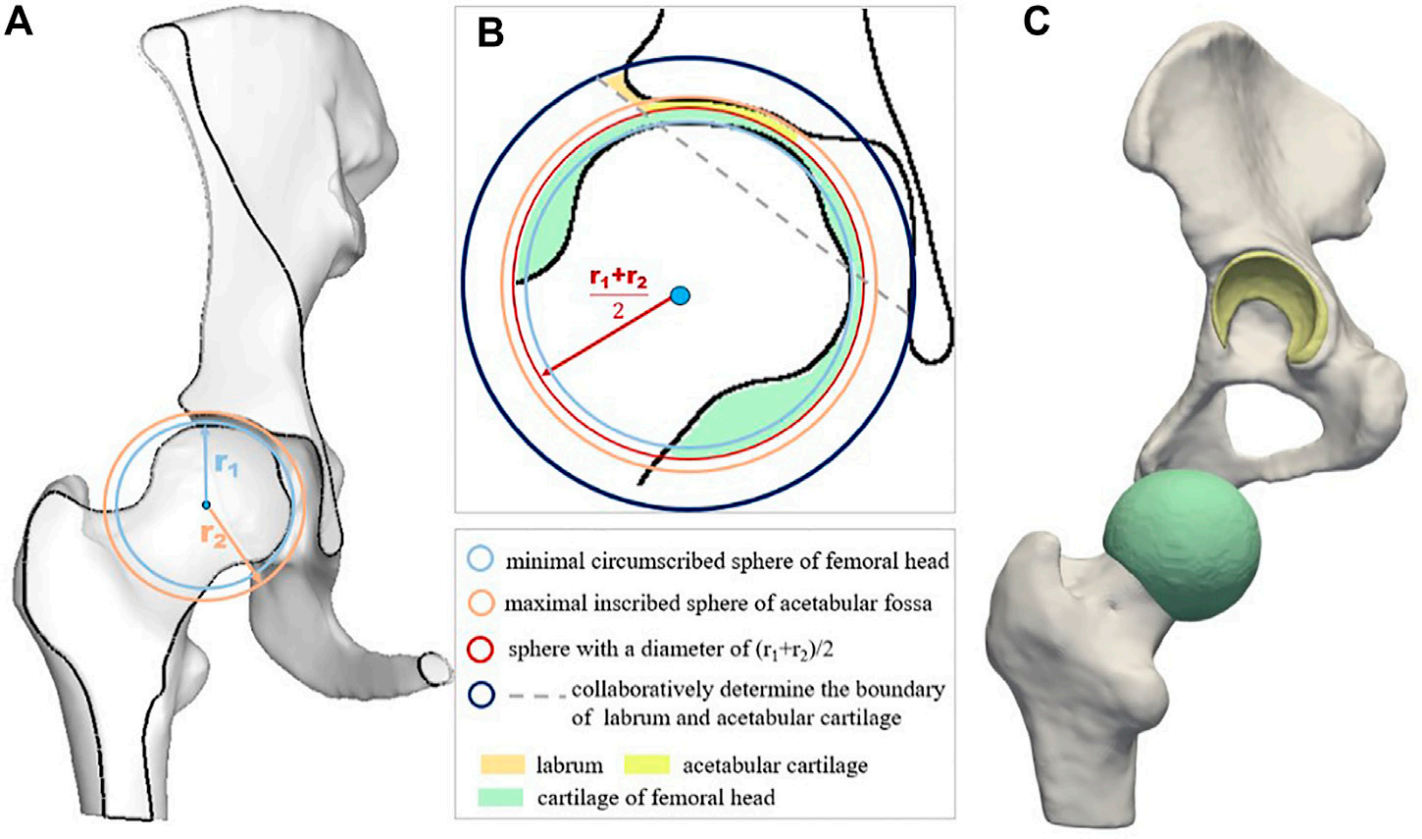
Schematic diagram of cartilage construction method including acetabular cartilage, cartilage of femoral head, and the labrum. **(A)** displays the minimal circumscribed sphere of the femoral head and the maximal inscribed sphere of the acetabular fossa; **(B)** is the schematic diagram of cartilage construction method; and **(C)** shows the geometric models of the cartilage layers.

The schematic diagram of the cartilage construction method is shown in Figure 2, and the cartilages included the acetabular cartilage, the cartilage of the femoral head, and the labrum. First, the center point ***O*** of the femoral head was calculated and used as the shared center point of the minimal circumscribed sphere (***S***_***A***_) of the femoral head with a radius of ***r***_***1***_, shown by the light blue circle in Figure 2A. Second, the maximal inscribed sphere of the acetabular fossa (***S***_***B***_) was built based on the center point *O*, and the corresponding radius was denoted as ***r***_***2***_ (Figure 2A, orange circle). The homocentric sphere ***S***_***C***_ with radius (***r***_***1***_ + ***r***_***2***_)/2 was generated. It was the interface boundary between the acetabular cartilage and the cartilage of the femoral head, as shown in the red circle in Figure 2B. The cartilage of the femoral head was then obtained by subtracting the femoral head volume using the sphere ***S***_***C***_ *via* the Boolean operation. Another homocentric sphere ***S***_***D***_ with a larger radius was established to build the labrum considering its high mechanical impact (Figure 2B, dark blue circle). The acetabular cartilage and labrum combination was calculated by ***S***_***D***_ minus ***S***_***C***_ and then cut by the dotted gray line concerning the labrum thickness and deleted the part connected to the acetabular fossa. The constructed femoral head cartilage and the acetabular cartilage and labrum were built and represented by green and yellow in Figure 2C.

### 2.3 Mesh Generation and Material Property Assignment

The reconstructed acetabulum and femoral head were discretized with tetrahedral grid in all of the hip models. The average cell numbers of the acetabular tetrahedral units, femoral tetrahedral units, and tetrahedral cartilage units were 251,068, 52,720, and 3,837, respectively. Grid independency analysis was conducted before the computations of all the hip models, which confirmed that the baseline grid assignment was adequate for the current research. Detailed information regarding the mesh validation is presented in the Supporting Document.

The contact parts between models were treated as common nodes. All discretized models were imported back to Mimics (Materialise) to assign material properties according to the spatial gray value in CT scans. The Young’s modulus of the cartilage tissue was set as 10.35 MPa (Ike et al., 2015), whereas that for the bone tissue was calculated based on the relationship between the element-specific gray value in the CT images and the elasticity property of the material (Lequesne, 1961b). The Poisson ratio of all bone tissues and cartilage tissues was set as 0.4. By applying the formulas presented in Table 2, the spatial material attributes could be assigned in the center of each element.

**TABLE 2:** Formulas describing the transformation from gray value to elastic modulus.

Gray value	Bone density (g/cm^3^)	Young’s modulus (MPa)
**HU** ≤ −1	ρ = 0	0.001
**HU** > −1	ρ = (HU + 1.4246) × 0.001/1.058	
	0 < ρ ≤ 0.27	33900ρ^2.20^
	0.27 < ρ < 0.6	5307ρ + 469
	0.6 ≤ ρ	10200ρ^2.01^

HU, Hounsfield units.

### 2.4 Boundary Conditions

**FIGURE 3:**
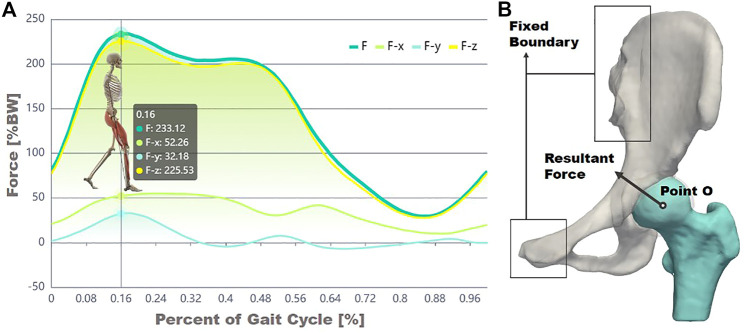
Experimental data of force decomposition over a gait cycle **(A)** and the illustration of forces and constraint boundary conditions **(B)**.

Previously, Bergeman et al. used sensors to detect the change in the direction of stress between the acetabulum and the femoral head of ordinary people in various activities of daily life (Figure 3A) (Bergmann et al., 2010). In the current study, the state in which the acetabulum experienced the most significant stress throughout gait was investigated. An averaged body weight of 60 kg was considered. Based on measurements of the curve in Figure 3A, the magnitudes of the load in the *X*, *Y*, and *Z* directions were 307.29, −189.22, and 1,326.12 N, respectively, (Bergmann et al., 2010), and the force acting site was at the center of the femoral head (Figure 3B). The sacroiliac joint and the pubic symphysis were set with constraint condition of zero displacements. The acetabular cartilage and the acetabulum, the femoral cartilage, and the femoral were set as the bound state. Considering that the nonlinear contact between the femoral head and the acetabulum might bring uncertainty to the entire solution process, this study used a constraint equation to constrain the contact surface between the two cartilages, transforming the nonlinear contact problem into a linear contact problem, in order to ensure the stability of the solution process.

### 2.5 Computation Modeling and Results Analysis

A finite element solver (ANSYS Inc., 2019) was applied to numerically solve the structural mechanics equations. According to the fourth strength theory (the von Mises theory or shape change–specific energy density theory), the von Mises equivalent stress was selected as the reference standard. The maximum stress values and quantitative stress distributions were analyzed by selecting regions of interest (ROIs). We mainly focused on the acetabulum and the contact surface of acetabular cartilage and acetabulum, that is, the facies lunata, through the equivalent stress program provided by the finite element solution.

## 3 Results

### 3.1 Global Acetabular Stress Analysis

**FIGURE 4:**
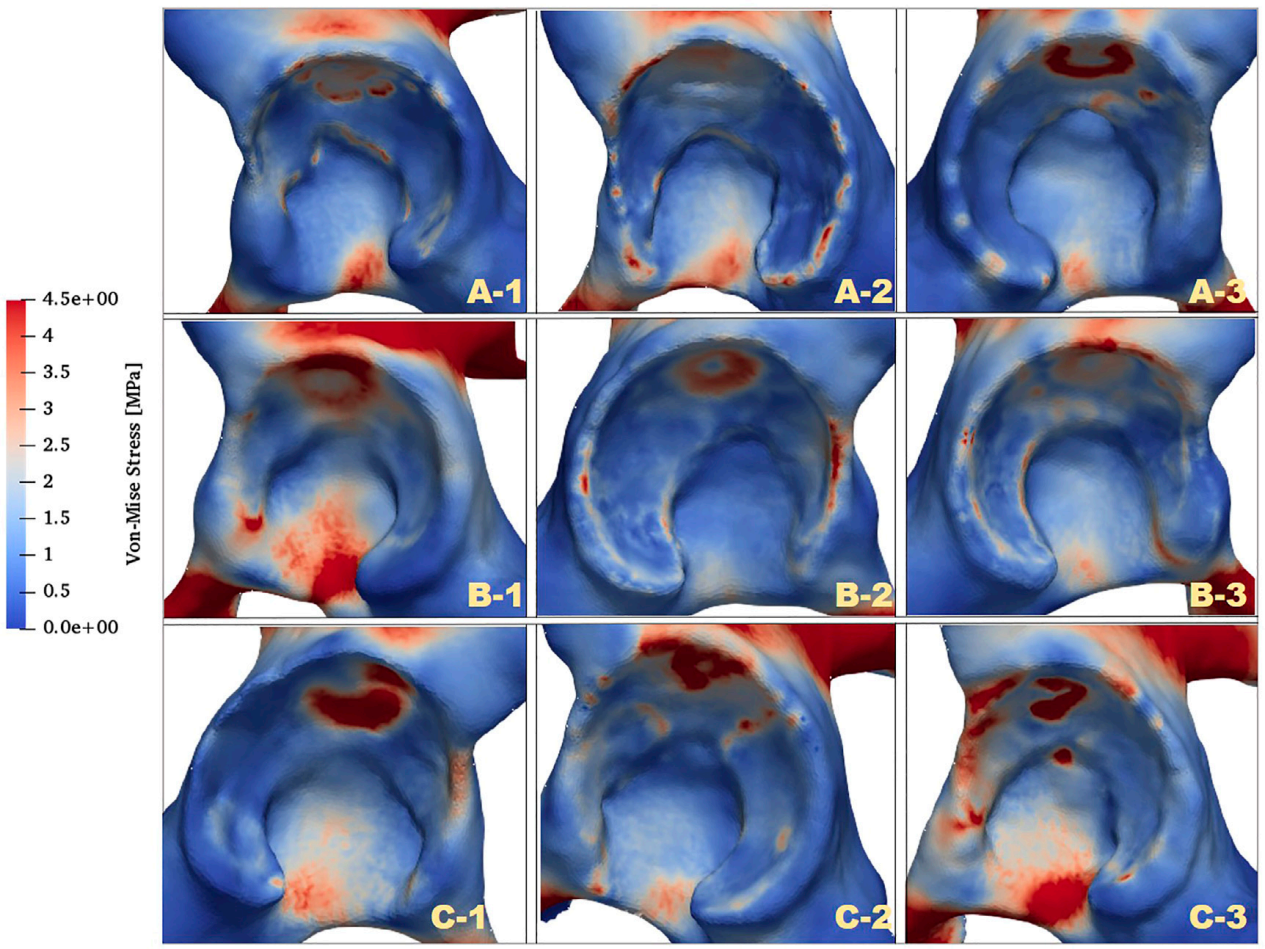
Comparison of stress distributions of the acetabulum at different LCEA and ACEA angles.

The distributions of von Mises stress (simplified as stress below) of the acetabulum in the three groups are plotted in Figure 4. In all cases, a high-stress area was located at the acetabular labrum with various degrees and positions. This phenomenon was consistent with the statement proposed by Hanak et al., which reported that the labrum in dysplastic hips supported more of the load transferred across the joint than in normal hips (Henak et al., 2011; Henak et al., 2014). With reduction of the ACEA, the high-stress concentration region was moving from the side near the acetabular fossa to the labrum side. For case C_1, the stress on the labrum was relatively low with a significantly smaller area compared with the other cases. This might be because the LCEA and ACEA of this model (LCEA of 20.1° and ACEA of 21°) were closer to the standard values than other models.

Apart from the stress distribution, the maximum stress of the acetabulum for all models was also extracted, and the highest stress magnitudes were listed in Table 3. It was clearly shown that the maximal stress value increased with the decline of ACEA in the same group. Notably, the maximum stress in group B was higher than that in groups A and C. The contact area between the acetabulum and acetabular cartilage was measured for all cases and recorded in Table 3. Results revealed that the average contact area in group B was 1,871 mm^2^, which was much lower than that in group A (2,224 mm^2^) and group C (2,209 mm^2^). Therefore, the relatively high stress in group B might be related to the smaller contact area.

**TABLE 3:** Quantitative stress analysis on acetabulum and local facies lunata.

Patients		A_1	A_2	A_3	B_1	B_2	B_3	C_1	C_2	C_3
**Maximum stress of the acetabulum [**MPa**]**		5.08	5.43	6.31	6.67	6.87	10.31	5.88	6.42	6.75
**Contact area [**mm^2^ **]**		2,236	2,416	2,021	1,763	2,047	1,830	2,352	2,287	1,990
**Area percentage [**%**]**	[0–1) MPa	72.57	61.2	49.64	75.4	82.1	76.07	73.67	52.07	38.64
	[1–2) MPa	22.47	30.6	32.03	19.64	14.02	19.68	17.63	32.27	38.25
	[2–3) MPa	4.41	7.5	9.17	3.67	3.18	3.92	5.15	9.43	19.92
	[3–4) MPa	0.48	0.6	3.36	0.9	0.58	0.12	2.04	3.47	6.62
	[4–5) MPa	0.05	0.09	3.57	0.29	0.12	0.14	0.96	1.92	2.61
	[5–6) MPa	0	0	2	0.11	0	0.05	0.55	0.59	0.8
	[6–7) MPa	0	0	0.24	0	0	0	0	0.24	0.14

### 3.2 Quantitative Stress Distribution of Facies Lunata

**FIGURE 5:**
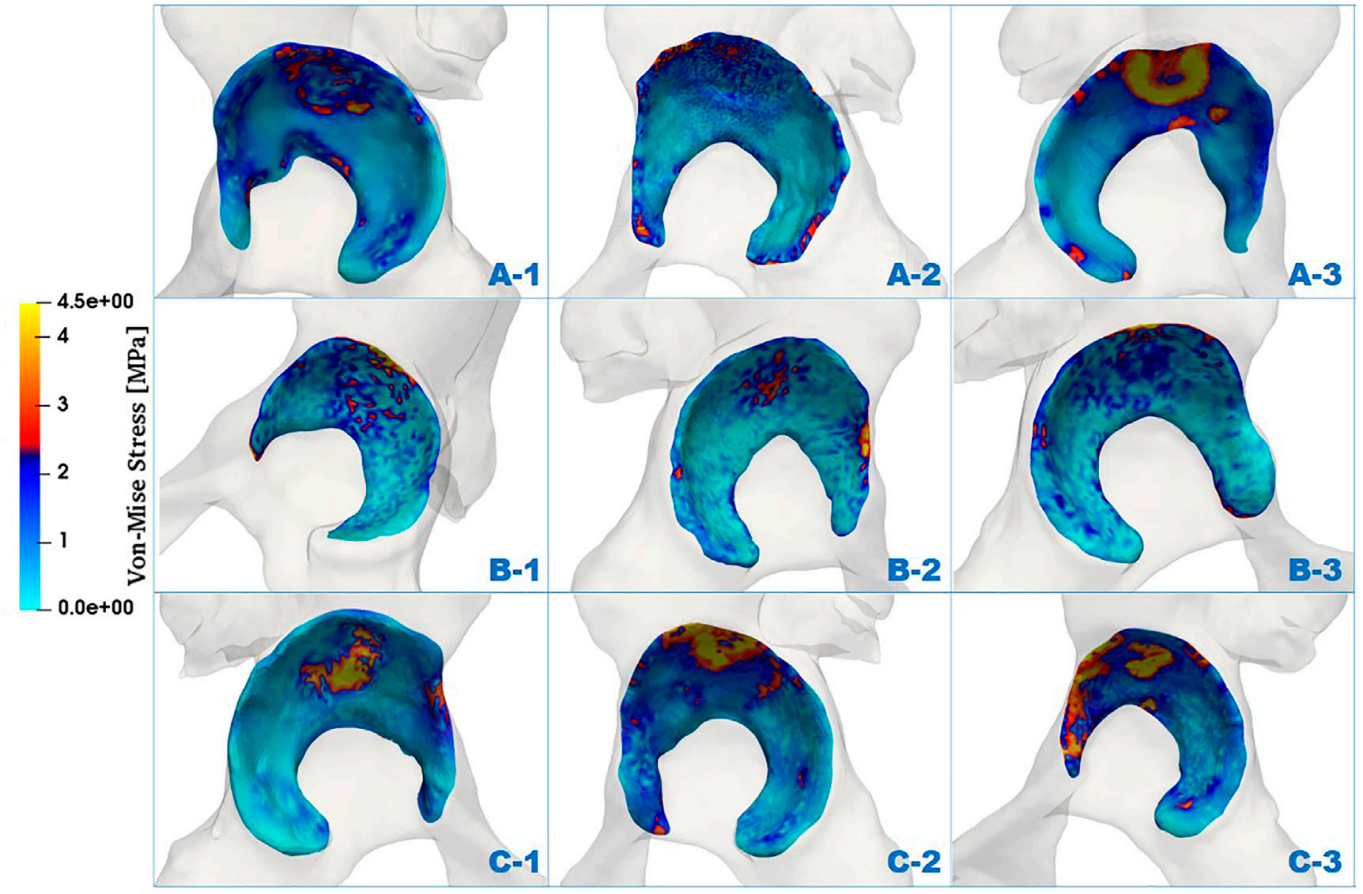
Comparison of von Mises stress distributions of facies lunata at different LCEA and ACEA angles for all models.

To further investigate the mechanics of the acetabulum, the stress distributions of the local facies lunata for each case were extracted, as shown in Figure 5. Compared with the stress distribution on the global acetabulum, Figure 5 displays that the high-stress area gradually moved toward the edge and increased on the local labrum with the decline of ACEA. This mechanical behavior was recognizable, especially in group C, where the area contact region decreased monotonic, indicating that the contact area played an essential role on the mechanical distribution. Furthermore, the extracted stress data were divided by an interval of 1 MPa, and the corresponding area ratio was calculated for each interval. The area ratio of each stress interval at different ACEAs was compared.

**FIGURE 6:**
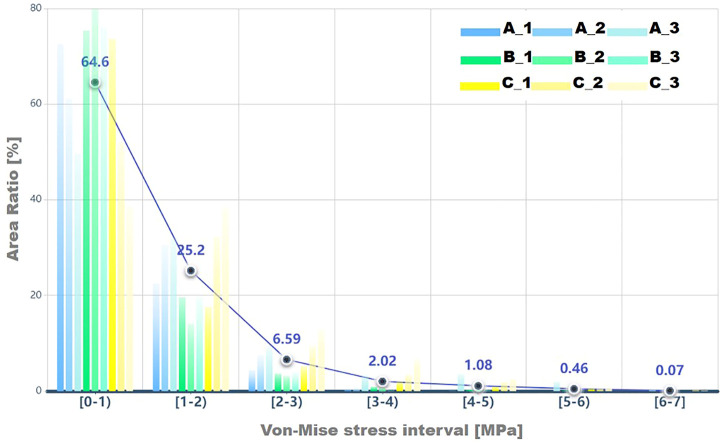
The area ratio in each von Mises stress interval for results on facies lunata is displayed, and a circle icon represents the average percent value for each stress interval.

The area ratios of each stress interval were calculated and are listed in Table 3 and shown as a histogram in Figure 6. Most stress values were located between 0 and 1, and the average number was 64.6% of all cases. Results presented a declined trend of area ratio with the rise of stress magnitude. Especially for the higher stress interval, that is, 5–7 MPa, the area percentage was significantly limited, which accounted for only a smaller proportion of the total area of the acetabular contact. The area decreased in groups A and C in the first interval, whereas it increased in other stress intervals as the ACEA declined, indicating the smaller ACEA leading to an incline to higher stress and corresponding larger area. In group B, there was no coincident tendency affected by the ACEA as shown in groups A and C. In fact, the area of the acetabular fossa of the B_1 model was 3,898 mm^2^, which was the smallest compared with the other models (≥4,000 mm^2^), and the B_1 model also had the lowest contact area (1,736 mm^2^), as shown in Table 3.

## 4 Discussion

Accurate diagnosis of hip dysplasia is of great significance for the subsequent decision-making of the treatment strategy, that is, conservation therapy or surgery. Current diagnosis and the severity evaluation mainly rely on the morphologic information, such as the LCEA, the ACEA, the Tönnis angle, and so on, which were provided by medical imaging. It has been reported that the LCEA could be considered as a reliable index because it is closely related to other radiographic markers and is highly associated with cartilage damage and osteoarthritis severity (Zhang et al., 2020). Moreover, it has been extensively used for the clinical diagnosis of DDH. However, when LCEA falls into the range of 18°–25°, physicians might face the challenge to precisely determine the dysplasia status and the severity. Therefore, to reveal other related morphological indicators and to understand the underlying mechanisms are needed. In this study, the influence of the ACEA on the mechanical effects of the hip was quantitatively studied. Together with LCEA, the results preliminarily confirmed the rationality of adding ACEA as the indication to refine the severity evolution of BDDH.

In order to obtain the stress variation accurately in the acetabulum with the change of ACEA angle, a new method was proposed in the operation of cartilage reconstruction. The thick articular cartilage layer in the hip joint helps to slow down the tremendous force passing through the hip joint, which can significantly influence the study of the mechanical behavior of the hip joint. Cartilage layer can be observed in magnetic resonance imaging (MRI) but not available in CT scans unless contrast is injected (Harris et al., 2012; Li et al., 2018; Wesseling et al., 2019). However, the majority of BDDH patients in outpatient experienced only the X-ray and ordinary CT scans. In this research, the cartilage was constructed based on the model reconstructed from CT images (Anderson et al., 2010), and resultant cartilage morphology was comparable to the data reported in previous studies (Ng, 2017). More importantly, a higher percentage of the load was transferred to the labrum in the dysplastic model because the femoral head achieved equilibrium near the lateral edge of the acetabulum (Henak et al., 2011). Therefore, we proposed a new acetabular cartilage construction method that combined the labrum into the cartilage (Section 2.2). Previous studies have demonstrated that the maximum stress value is mainly concentrated in the middle and upper part of the lunar surface of the acetabulum (Zhao et al., 2010; Ghosh et al., 2015; Liu et al., 2015). Our results revealed that the labrum region presented a concentrated high-stress area. This area moved toward the edge side with the decrease in ACEA, indicating that the labrum suffered from increasing stress at a smaller ACEA. This was consistent with the measurement conducted by Henak et al. (2011), who suggested that the labrum played a more significant role in load transfer and joint stability in hips with acetabular dysplasia than in hips with standard acetabular geometry. A more detailed comparison had been made and attached in the Supporting Document. This confirmed the rationality of the current model and the reported mechanical distribution in this study. As to the bone material, we spatially assigned anisotropic material characteristics based on the Hounsfield unit values in CT images, which was an advantage compared with the uniform assignment of the bone material (Dopico-González et al., 2010; Ghosh et al., 2012). According to the force magnitude and direction of the hip joint in the whole gait range obtained by Bergmann et al. (2010), the maximum contact force of the hip joint in the single supporting phase of normal walking mode was selected as the load condition of this study, to simulate the mechanical conditions in the ROIs in the acetabulum.

By solving the finite element models of the hip joint, the qualitative and quantitative analyses were performed on the global acetabulum and the contact region between the acetabulum and acetabular cartilage locally. Results showed that the maximum stress on the acetabulum increased as the ACEA decreased. Furthermore, quantitative analysis on the contact region revealed that the area ratio of higher stress intervals (>2 MPa) increased with the decline of ACEA. These two key results presented a good agreement in groups A and C. However, they did not fully fit in group B. Combined with the geometric analysis, the higher maximum stress in the B_3 model was postulated because of the smaller ACEA and the lower area of facies lunata. The area ratio in the range of 0–2 MPa was also smaller for the B_1 model, with a maximum ACEA in group B. It should be noted that the B_1 model presented the smallest area of both facies lunata acetabular fossa among all groups. This extraordinary phenomenon in group B suggests that, apart from the ACEA angle, the surface area of facies lunata and acetabular fossa was also an important factor in the mechanical distribution of the hip joint.

This study also implicated that the finite element model could be used as a valuable tool to assist the evaluation of the severity of BDDH. The contact area of BDDH was mainly limited to the upper half, and the stress concentration area was also in the upper part of the acetabulum, which was similar to the prediction by Anderson et al. (Kaku et al., 2004). However, a portion of the acetabular concentration predicted by our analysis was located in the acetabular fossa because of the dysplastic acetabulum poor coverage of the femoral head.

The present study suffered from several limitations, which should be further improved in the future. First, the impact of the Tönnis angle was not considered, which might also play a role in the acetabular mechanical behavior. Because the LCEA, ACEA, and Tönnis angle were interdependent, the idealized model was not applicable in the current study. Because of the strict inclusion criteria, the influence of the Tönnis angle could not be studied in the current research. Second, the cartilage construction was not established based on MRI or enhanced CT. The modified reconstructed methods were proposed by this study, which was able to generate relatively realistic models of the cartilage based on ordinary CT images. Future studies could be improved when multimodality data were available for each patients. Third, a single loading state was analyzed in the current study. Dynamic analysis of a complete gait cycle with various load patterns should be achieved in the future.

## 5 Conclusion

This study investigated the relationship between ACEA and the mechanical behavior of the acetabulum of BDDH. By establishment of comprehensive finite element models of the hip joint, this study revealed that the high-stress concentration area increased as the ACEA declined. The contact region of facies lunata was also a significant factor influencing the stress distribution. This research confirmed the effectiveness of the ACEA in determining the severity of BDDH, which might facilitate precise evaluation of the disease when LCEA was in the range of 18°–25° and thus contribute to wise decision-making of the subsequent treatment.

## Data Availability

The data that support the findings of this study are available on request from the corresponding authors. They are not publicly available due to privacy or ethical restrictions.
