# Supplementary material for: Role of the Anterior Center-Edge Angle on Acetabular Stress Distribution in Borderline Development Dysplastic of Hip Determined by Finite Element Analysis
Source: Front Bioeng Biotechnol. 2022 Mar 1;10:823557. doi: 10.3389/fbioe.2022.823557 (PMC8921530; doi:10.3389/fbioe.2022.823557)
Supplement: Supplementary file 1 [file DataSheet1.docx]

**Supporting Document**

**Role of the Anterior Center-edge Angle on Acetabular Stress Distribution in Borderline Development Dysplastic of Hip determined by Finite Element Analysis**

Songhao Chen^1,#^, Liqiang Zhang^2,3,#^, Yuqian Mei^1,4,^,Hong Zhang^5^, Yongcheng Hu^6,*^ , Duanduan Chen^1,*^

^1^ School of Life Science, Beijing Institute of Technology, China

^2^ Tianjin Medical University, Tianjin, China

^3^ Department of Orthopaedics, Shanxi Children's Hospital, Taiyuan, China

^4^ School of Medical Imaging, North Sichuan Medical College, Sichuan, China

^5^ Department of Orthopaedics, The Fourth Medical Centre of PLA General Hospital, Beijing, China

^6^ Department of Bone and Soft Tissue Oncology, Tianjin Hospital, Tianjin, China

**^#^** Songhao Chen and Liqiang Zhang are co-first authors.

**I. Grid independency study**

To confirm the insensitivity of the results to the spatial resolution of the grid, the grid independency analysis was conducted. Apart from the base discretization, the solution on a finer grid with 253,401 cells (model-A) and 505,842 cells (model-B) have also been investigated, which were about 5-times and 10-times finer than the baseline model. To compare the results of these three discretizations, the maximum equivalent stress on the femoral surface while standing, shown in Figure S1(A), was studied. Loading conditions were assigned with the same method described in the manuscript, as shown in Figure 1S(B).

The exact stress distribution pattern was found among the three models and the maximum equivalent stress obtained by the numerical simulation of the three models was compared and shown in Table S1. The discrepancies of the maximum equivalent stress between the three grids were 3.9% (model-A to baseline model) and 3.7% (model-B to baseline model). Therefore, for the purposes of our study the base resolution of 52,720 cells of the femur was considered adequate.


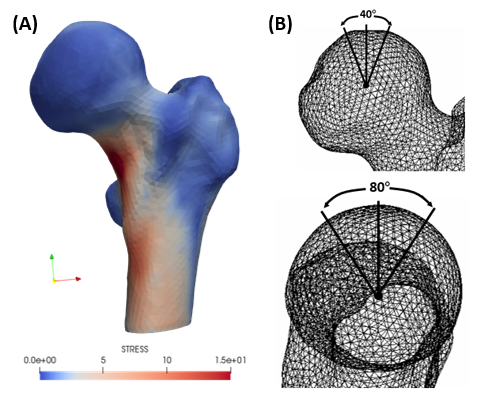


**Figure S1** Distribution of the equivalent stress of the femur moel (A) and the loading site of the contact force of the proximal femur hip joint (B).

**Table S1** The maximum equivalent stress of mesh analysis models

| Model | The maximum equivalent stress (MPa) |
| --- | --- |
| Baseline model | 17.522 |
| Model-A | 18.268 |
| Model-B | 18.170 |

**II. Comparison to the literature**

Table S2 summarized the comparison of our research with other literature of the computational studies of hip joint in recent years. Our research focused on borderline hip dysplasia, which is becoming a hot issue in the hip field in recent years. More specifically, the current study investigated the influence of anterior center-edge angle (ACEA) on the mechanical stress distribution of the hip joint, via finite element analysis. Compared with other literatures, the cartilage reconstruction method has been modified and improved and stress distribution, especially on the contact interface of the hip joint was reported. By comparing the stress analysis with the previous literatures, the rationality of this study was verified. Details of the comparison were listed in Table S2.

**Table S2** Comparison to the related studies

|  | Biomechanical features of the hip | | Disease/  Study purpose | Methods for cartilage reconstruction | Year/  References |
| --- | --- | --- | --- | --- | --- |
|  | The stress on the labrum | The concentrate stress of the acetabulum (distribution) |  |  |  |
| 1 | N/A | The load-bearing area | DDH/  Sagittal pelvic tilt | Uniform thickness cartilage  modeling method | 2021/[1] |
| 2 | N/A | The anterosuperior and superior acetabulum | DDH/  Sagittal pelvic tilt | Uniform thickness cartilage  modeling method | 2020/[2] |
| 3 | N/A | The superior and anterior rigion | DDH/  Femoroacetabular impingement | N/A | 2019/[3] |
| 4 | N/A | Margin | DDH/  Shelf acetabuloplasty | N/A | 2018/[4] |
| 5 | N/A | The superior-posterior region | DDH/  Acetabular defects | N/A | 2018/[5] |
| 6 | N/A | The superolateral region | DDH/  PAO | Uniform thickness cartilage  modeling method | 2018/[6] |
| 7 | N/A | The supra-acetabular region | SAIFs | Construction based on MRI | 2018/[7] |
| 8 | The load was transferred to the labrum. | The middle and upper part | Healthy subjects/  Cartilage mechanics | Combined cartilage layer method | 2015/[8] |
| 9 | The labrum in dysplastic hips supported 2.8-4.0 times more of the load transferred across the joint than in normal hips. | The primary load-bearing regions | DDH/  Cartilage mechanics | Construction by injection of contrast | 2014/[9] |
| 10 | The labrum in the dysplastic model supported 4-11% of the total load transferred across the joint, while the labrum in the normal model supported only 1-2% of the total load. | N/A | DDH/  Acetabular labrum | Construction by injection of contrast | 2011/[10] |
| 11 | A higher percentage of the load was transferred to the labrum. | The anterior and upper acetabulum (with reduction of the ACEA, the high-stress concentration region was moving from the side near the acetabular fossa to the labrum side) | BDDH/  ACEA | A modified numerical method | Our study |

DDH, Developmental dysplasia of the hip

BDDH, Borderline development dysplastic of the hip

PAO, Periacetabular osteotomy

SAIFs, Supra-acetabular insufficiency fracture

MRI, Magnetic resonance imaging

1.Hasegawa K, Kabata T, Kajino Y, Inoue D, Tsuchiya H: **The Influence of Pelvic Tilt on Stress Distribution in the Acetabulum: Finite Element Analysis**. 2020.

2.Kk A, Mf A, Tu A, Mi A, Si A, Sh A, Gm A, Mt B, Yn A: **Effect of sagittal pelvic tilt on joint stress distribution in hip dysplasia: A finite element analysis**. *Clinical Biomechanics* 2020, **74**:34-41.

3.Diaz-Lopez RA, Alonso-Rasgado MT, Jimenez-Cruz D, Bailey CG, Board TN: **Impact of femoroacetabular impingement and dysplasia of the hip on hip joint sphericity**. *Hip International* 2019, **30**(2):112070001983429.

4.Kaga N, Iwami T, Saito K, Akira K, Shimada Y: **Finite Element Analysis of the Efficacy of Shelf Acetabuloplasty for Acetabular Dysplasia**. *International Journal of Physical Medicine & Rehabilitation* 2018, **06**(6).

5.Xiao J, Zhao X, Wang Y, Yang Y, Zhao J, Gao Z, Zuo J: **Application of acetabular reinforcement ring with hook for correction of segmental acetabular rim defects during total hip arthroplasty revision**. *Journal of Bionic Engineering* 2018, **15**(1):154-159.

6.Sung-Jae P, Sung-Jae L, Chen WM, Jung-Hong P, Yong-Soo C, Taejin S, Soon-Yong K: **Computer-Assisted Optimization of the Acetabular Rotation in Periacetabular Osteotomy Using Patient's Anatomy-Specific Finite Element Analysis**. *Applied Bionics and Biomechanics,2018,(2018-2-4)* 2018, **2018**:1-11.

7.Hidetatsu T, Go Y, Hiroaki K, Shutaro Y, Yu M, Daisuke C, Etsuo C, Eiji I: **Biomechanical analysis of supra-acetabular insufficiency fracture using finite element analysis**. *Journal of Orthopaedic ence* 2018, **23**:825-833.

8.Ghosh R, Pal B, Ghosh D, Gupta S: **Finite element analysis of a hemi-pelvis: the effect of inclusion of cartilage layer on acetabular stresses and strain**. *Comput Methods Biomech Biomed Engin* 2015, **18**(5-8):697-710.

9.Henak CR, Abraham CL, Anderson AE, Maas SA, Ellis BJ, Peters CL, Weiss JA: **Patient-specific analysis of cartilage and labrum mechanics in human hips with acetabular dysplasia**. *Osteoarthritis and Cartilage* 2014.

10.Henak CR, Ellis BJ, Harris M, Anderson AE, Peters CL, Weiss JA: **Role of the acetabular labrum in load support across the hip joint**. *Journal of Biomechanics* 2011, **44**(12):2201-2206.
